# Primary Spinal Astrocytomas: A Literature Review

**DOI:** 10.7759/cureus.5247

**Published:** 2019-07-26

**Authors:** John Ogunlade, James G Wiginton, Christopher Elia, Tiffany Odell, Sanjay C Rao

**Affiliations:** 1 Neurosurgery, Riverside University Health System Medical Center, Moreno Valley, USA; 2 Neurosurgery, Desert Regional Medical Center, Palm Springs, USA; 3 Neurosurgery, Kaiser Permanente - Fontana Medical Center, Fontana, USA

**Keywords:** astrocytoma, intradural, intramedullary, spinal astrocytoma, spinal cord neoplasms, glioblastoma, glioma, spinal tumor

## Abstract

Primary spinal astrocytoma is a subtype of glioma, the most common spinal cord tumor found in the intradural intramedullary compartment. Spinal astrocytomas account for 6-8% of all spinal cord tumors and are primarily low grade (World Health Organization grade I (WHO I) or WHO II). They are seen in both the adult and pediatric population with the most common presenting symptoms being back pain, sensory dysfunction, or motor dysfunction. Magnetic Resonance Imaging (MRI) with and without gadolinium is the imaging of choice, which usually reveals a hypointense T1 weighted and hyperintense T2 weighted lesion with a heterogeneous pattern of contrast enhancement. Further imaging which may aid in surgical planning includes computerized tomography, diffusion tensor imaging, and tractography. Median survival in spinal cord astrocytomas ranges widely. The factors most significantly associated with poor prognosis and shorter median survival are older age at initial diagnosis, higher grade lesion based on histology, and extent of resection. The mainstay of treatment for primary spinal cord astrocytomas is surgical resection, with the goal of preservation of neurologic function, guided by intraoperative neuromonitoring. Adjunctive radiation has been shown beneficial and may increase overall survival. The role of adjunctive chemotherapy is employed, however, its benefit has not been clearly defined. Primary spinal cord astrocytomas are rare and challenging to treat. The gold standard treatment is surgical resection. Second-line treatments include radiation and chemotherapy, although, the optimal regimen for adjunctive therapy has not yet been clearly defined.

## Introduction and background

Epidemiology

Tumors of the spinal cord are rare and are more commonly seen in the pediatric population rather than in adults. In general, these tumors arise in one of three compartments of the spinal cord: extradural, intradural extramedullary, and intradural intramedullary. Of those tumors that arise in the intradural intramedullary compartment, spinal cord gliomas account for approximately 80% in all age groups [1,2]. Among spinal cord gliomas, astrocytomas comprise 30 to 40% and ependymomas comprise 60 to 70% [1,3]. The majority of intramedullary tumors are astrocytic or ependymal, however, hemangioblastoma (3-8%), ganglioglioma, lymphoma, and melanoma (rare) also contribute to the general epidemiological picture [1,2].

Subtypes of astrocytomas include pilocytic astrocytoma, diffuse astrocytoma, anaplastic astrocytoma, and glioblastoma multiforme (GBM, also known as grade IV astrocytoma or malignant glioblastoma). Primary spinal astrocytomas constitute approximately 6-8% of all spinal cord tumors, with primary spinal GBM comprising approximately 1.5% of spinal cord tumors [4,5]. In general, 75% of primary astrocytomas of the spinal cord are low-grade (WHO grade I and II) and are overall less aggressive when compared to primary astrocytomas of the brain [6,7]. The remaining 25% are high grade (WHO grade III and IV) lesions [7].

It is estimated that the incidence of spinal cord gliomas is 0.22 per 100,000 persons per year [3]. Anywhere from 850 to 1700 new cases of primary spinal cord gliomas are diagnosed each year in the United States [6]. In a survival-analysis of 459 adults with primary intradural intramedullary spinal cord tumors, 92.8% of the tumors were low-grade (WHO I or II) while 7.2% were high-grade (WHO III or IV), and the associated median survival was 10.2 years [8]. A genetic association has been found with neurofibromatosis type 1 and astrocytomas, which are more prevalent in males, and commonly present as low-grade astrocytomas in children and high-grade astrocytomas in adults. However, they are rarely seen above the age of 60 [9]. Patients with spinal astrocytomas commonly present with non-mechanical back pain, dysesthesias, paraesthesias, or motor dysfunction [10].

Many current reviews covering spinal cord gliomas focus on other intramedullary spinal cord tumors and gliomas. The objective of this review focuses specifically on literature related to primary spinal cord astrocytomas in the adult population and a summary of current and future treatment strategies.

## Review

Imaging

Magnetic resonance imaging (MRI) with and without gadolinium is the imaging modality of choice for spinal astrocytoma [11]. Spinal astrocytomas are usually located in the cervical (49%) and thoracic (67%) spine with some overlap when both segments are involved [12,13].

**Figure 1:**
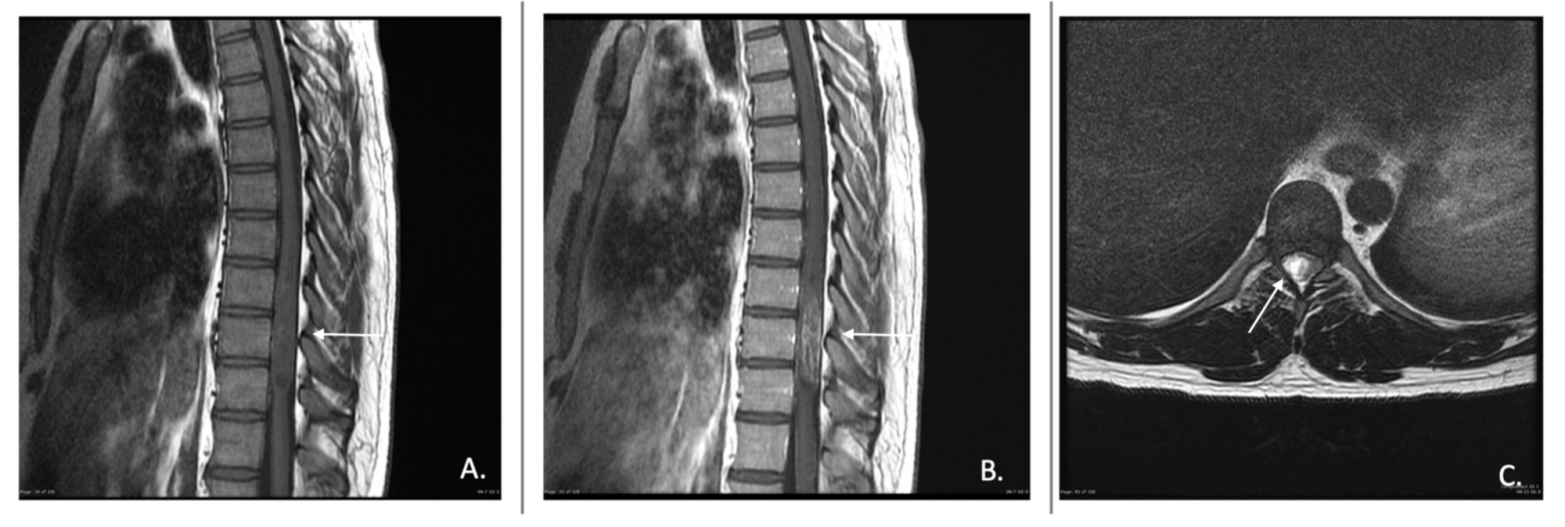
Pilocytic astrocytoma of the thoracic spine. A) T1-weighted sagittal pre-contrast image, B) T1-weighted sagittal post-contrast image, and C) T2-weighted axial slice showing displacement of thoracic cord by area of hyperintensity consistent with spinal astrocytoma. Photo Reference: Courtesy of Assoc. Prof. Frank Gaillard, Radiopaedia.org, rID: 19536

**Figure 2:**
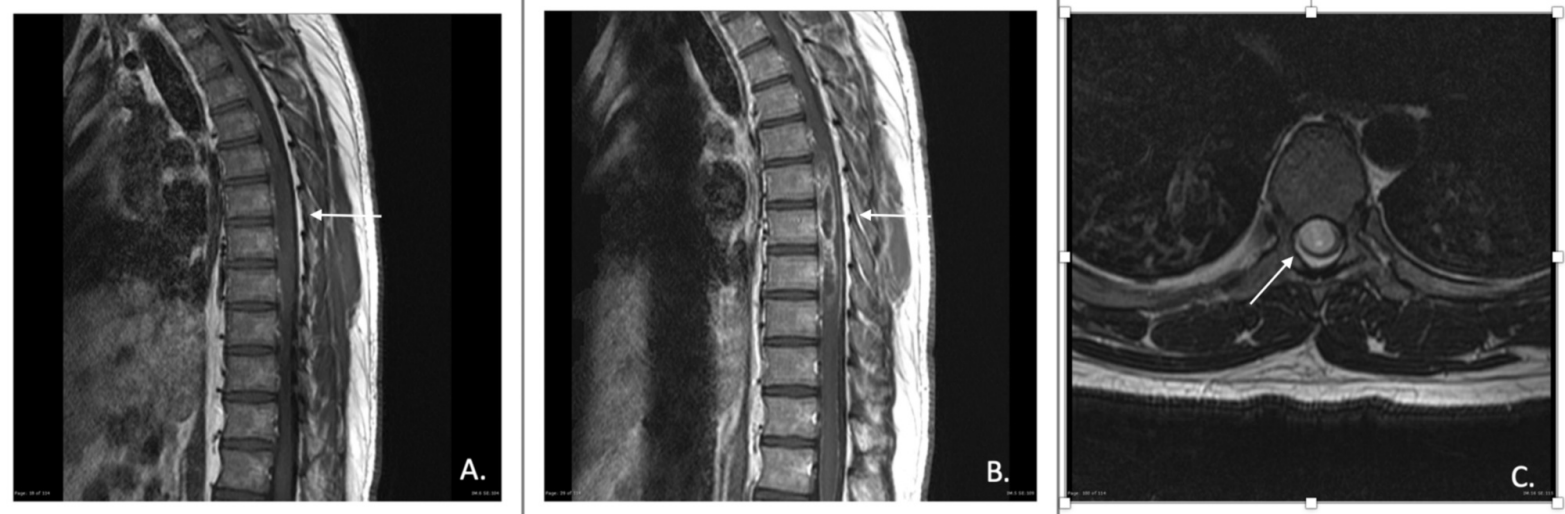
High-grade astrocytoma of the thoracic spine. A) T1-weighted sagittal pre-contrast image, B) T1-weighted sagittal post-contrast image, and C) T2-weighted axial slice showing displacement of the thoracic cord by area of hyperintensity consistent with spinal astrocytoma. Photo Reference: Courtesy of Assoc. Prof. Frank Gaillard, Radiopaedia.org, rID: 19536

On computerized tomography (CT), astrocytomas may be associated with increased interpedicular distance and bone erosion, however, these imaging findings are also commonly seen with ependymomas [14]. On MRI, astrocytomas are typically isointense or hypointense on T1 weighted imaging (T1WI) and hyperintense on T2 weighted imaging (T2WI) (Figures 1, 2). A vast majority of astrocytomas show some level of enhancement on postcontrast imaging. Cysts are a common finding of spinal astrocytomas. These cysts are usually intratumoral and have peripheral contrast enhancement [15]. About 57% of astrocytomas are eccentric in the cord, due to their origination from the cord parenchyma, therefore, may create a focal expansion of the spinal cord diameter or cause displacement of normal spinal parenchyma [12].

A retrospective review of 19 spinal cord astrocytomas found enhancement in 68% of tumors; typically in a focal nodular, patchy, and inhomogeneous pattern with none exhibiting diffuse homogeneous enhancement. This pattern of enhancement aids in distinguishing astrocytomas from ependymomas of the spinal cord, and gives a clue to a more infiltrative process [16]. However, a multicenter retrospective study found that one-third of astrocytomas in its cohort did not show any enhancement on post-contrast MRI, suggesting that lack of enhancement does not always correlate with the level of invasion [16].

 

Diffusion tensor imaging (DTI) and diffusion tensor tractography (DTT) have been utilized in surgical planning in patients with spinal cord astrocytomas to mitigate the morbidity associated with surgery; however, their efficacy is debated. Some advocate that magnetic resonance (MR) DTI and DTT are unreliable in tumors with cystic components and more reliable for solid tumors based on anecdotal clinical series [17].

In a more recent study, DTI and DTT were used on a cohort of eleven patients with spinal cord astrocytoma and were found to be more beneficial in assessing the extent of tumor involvement of the spinal cord due to the ability to obtain axial views. Based on DTT, the authors categorized the 11 patients based on the involvement of the spinal cord, and thus developed a classification scheme. Astrocytomas of the cervical cord were divided into infiltrating type (type I) and displacement type (type II). Type I tumors were further subdivided into simple infiltrating (Type IA) and infiltrating with destruction (Type IB). One patient with a solid mass, two with cystic degeneration, and one with peritumoral cyst were classified as type II. Such a classification, suggested for the first time, might have important implications on further treatment and analyzation of these tumors [18].

Risk factors and prognosis

Age, histological diagnosis (morphology/WHO grade), preoperative neurological function, and period of diagnosis are all predictors of the outcome [3,8,19-21]. However, age greater than 60 at time of diagnosis, high-grade morphology, and extent of resection have the most significant impact on overall survival [4]. In a study following 19 consecutive cases of grade III and IV spinal astrocytomas in a younger population (median age 14), the median postoperative survival was only six months after undergoing a combination of radiation therapy and radical surgical resection [22].

Wong *et al.* also investigated risk factors and overall survival for patients with spinal cord astrocytomas. Their univariant analysis suggests that male gender, the extent of surgical resection, and tumor histology were significant predictors of survival. Male patients had double the mean survival versus female patients (24 months versus 12 months) suggesting some level of hormonal or genetic influence. These studies consistently record poor prognostic correlation with higher-grade tumors, with spinal GBM having a significantly higher rate of mortality [23]. Results from a cohort of 664 patients with spinal astrocytomas showed that the five-year overall survival rate was 82% for grade 1, 70% for grade 2, and declined to 28% and 14% for grade 3 and 4, respectively [3].

At least 165 primary spinal GBM cases have been reported since 1938 with a slight male preponderance. They are more prevalent in young individuals (mean age 26 years); found typically in the thoracic or cervical region and least frequently in the conus medullaris [24]. The average survival is 14.3 months; however, age at the time of diagnosis weighs heavily on median overall survival. Patients diagnosed over the age of 50 years had a median overall survival of two months, and those diagnosed at less than 50 years of age had a median overall survival of 14 months [25]. Other reviews of high-grade gliomas (grade III and IV) report an 18.7-month post-surgery survival but also quote a low likelihood of gross total resection (approximately 10%) [10].

A case series of 25 adult patients who underwent gross total surgical excision of spinal astrocytomas also found that the tumor histology affected the overall outcome most significantly. Five of six patients with grade IV astrocytoma died within 23 months from surgery, while 17 of 19 patients with low-grade lesions (grade I or II) had a mean survival of 50.2 months [26]. In an institutional review of six patients with spinal cord GBM, the functional status exhibited uniform deterioration over one year [27].

A retrospective study of 89 adult patients with primary malignant spinal cord astrocytoma (44 grade III and 45 grade IV) found that gross total resection, when possible, led to significantly lower mortality when compared to subtotal resection, biopsy, or non-surgical treatment [19]. In this retrospective study, the reader should consider that the reported gross total resections may be the result of favorable surgical planes of dissection, which may indicate a less infiltrative tumor and, therefore, a better survival. 

Approximately 20 reports of spinal GBM with secondary cerebral manifestation have been identified. High-grade primary intracranial tumors with secondary intraspinal manifestation are more commonly seen and portend a worse overall survival of 4.2 months according to recent studies [25,28].

Treatment

Surgery

Surgical resection and decompression is the optimal treatment strategy for spinal cord astrocytomas [10,24,29-31]. In non-disseminated yet malignant astrocytomas, aggressive resection generally leads to increased overall survival [30]. However, gross total resection in high-grade (grade III and IV) astrocytomas is often not feasible with some reports citing gross total resection being possible in 0% of their grade IV lesions and only 12% of their grade III lesions [5]. In a retrospective review of 22 patients with high-grade intramedullary astrocytomas, only two achieved a gross total resection [10]. Another study outlines the morbidity associated with attempted gross total resection (GTR) of high-grade spinal cord astrocytomas: 37% of 46 patients had a worse neurologic function as compared to before surgery [32]. Overall, gross total resection should be pursued if there are good planes of dissection intra-operatively and if there is stable neuromonitoring throughout the case irrespective of tumor grade [33]. 

Intraoperative neuromonitoring for intramedullary spinal cord tumors is of paramount importance. A combination of muscle motor evoked potentials (mMEP), and direct wave (D-wave) monitoring should be used as the gold standard for allowing for the most aggressive resection possible while monitoring for neurologic changes [34]. Expansile duraplasty is commonly performed and recommended as the safest option to allow for maximal decompression [35].

Radiation Therapy

In a retrospective analysis of 16 patients receiving radiotherapy for spinal cord glioma (with ependymoma excluded), radiation therapy was found to be an ineffective primary method of treatment as the mean overall survival was 2.7 months. In contrast, patients who received surgery before radiation therapy had an overall survival of 64 months. The median radiation dose was 45 Gray (Gy), and the median dose per fraction was 1.8 Gy with a total radiation dose of greater than 45 Gy being optimal for providing improved overall survival [25].

In a study of 183 patients treated with either surgery alone or surgery plus radiation, it was found that postoperative radiation therapy was effective at reducing disease progression in low and moderate grade (grade I and II) astrocytomas [36].

It is important to note that radiation therapy comes with its inherent risks. A study by Li et al. in rat models shows endothelial apoptosis of the spinal cord within 24 hours of radiation to the cord [37]. Despite these inherent risks, radiation therapy remains the standard of care for patients who have undergone surgical biopsy or resection and have a confirmed diagnosis of spinal cord astrocytoma. Radiation is not recommended as initial therapy for newly diagnosed spinal cord astrocytoma, but when used after surgery as adjunctive therapy, the overall survival is increased [25]. Radiotherapy is also an integral part of the management of recurrent astrocytomas [7].

Radiation-induced spinal cord GBM has also been reported [38]. Mutations in several human proteins and genes have been implicated in the development of spinal cord astrocytomas. These proteins include tumor suppressor protein p16, phosphate and tensin homolog protein (PTEN), and p53. Genes with known implication are BRAF and H3F3A. Overall, studies are limited, and most of the research stems from studies based on cranial astrocytomas [39]. 

Chemotherapy 

The role of chemotherapy remains debated, and its place in the management of patients with spinal cord tumors remains questionable. However, some advocate that chemotherapeutic agents used in astrocytomas of the brain can be utilized in spinal cord astrocytomas and that refractory astrocytoma (resistant to surgery and post-operative radiation) may be an especially ripe area for management with chemotherapy [7]. The combination of procarbazine, lomustine, and vincristine (PCV) has been reported to be of benefit in some cases. Henson et al. describe a case of low-grade spinal cord astrocytoma refractory to radiation and chemotherapy with cisplatin and etoposide after biopsy, which responded to treatment with PCV with a 23-month progression-free survival [40]. 

In a multi-institutional retrospective study of 22 adult patients with recurrent grade II and grade III astrocytomas initially treated with surgery and radiation therapy, treatment with temozolomide was effective in providing two years of progression-free survival. The probability of survival observed in this cohort was 64% at six months, 64% at 12 months, 41% at 18 months, and 27% at 24 months [41].

In conclusion, chemotherapy is currently most commonly used for refractory spinal cord astrocytomas, but its routine use as an adjunctive treatment is on the rise. Chemotherapy with or without radiation after surgery is an area of potential interest and future research.

Combination Therapy

Spinal cord GBM treatment typically consists of surgery, radiation, and chemotherapy. Opinions are mixed on whether GTR is beneficial in these patients as some reviews have quoted that GTR is feasible in 0-12.7% of patients [24]. However, adjuvant radiation therapy can prolong survival. Shen et al. report that in grade IV astrocytomas, gross total resection with chemotherapy and radiation therapy yields the same mean survival as subtotal resection, chemotherapy, and radiation therapy, 18.9 months versus 18.6 months [24]. Surgery and radiation without chemotherapy had a mean survival of 11.2 months for GTR and 12.8 for subtotal resection. The role of adjuvant chemotherapy in this patient population remains debated and controversial; however, studies suggest a potential benefit and further research on this topic is needed [24].

Raco et al. found that patients with high-grade astrocytomas (grade III and IV) who received radiotherapy plus chemotherapy after surgery had a mean survival almost 12 months longer than those undergoing surgery alone [10]. Likewise, the combination of fractionated radiation, temozolomide, and bevacizumab is used routinely in some institutions to treat spinal GBMs [27].

Novel Treatment Strategies

Ropper et al. investigated the efficacy of dual gene-engineered human neural stem cells in rats injected with human glioblastoma cell lines receiving both 5-fluorocytosine and ganciclovir for five days. In vitro and in vivo, it was found that this dual-gene therapy was superior to monotherapy or control group. There was an 83% inhibition of tumor cell proliferation as compared to 61% in monotherapy with 5-fluorocytosine. Post-mortem analysis showed incorporation of therapy cells into the tumor. This study demonstrates the potential ability of dual-gene therapy in the treatment of glioblastoma of the spinal cord [29].

## Conclusions

Primary spinal cord astrocytomas are uncommon, comprising only 6-8% of all spinal cord tumors. Treatment remains a difficult task. The associated morbidity and mortality are multifactorial. Studies have demonstrated that poor overall survival is primarily influenced by high-grade morphology, advanced age, and the feasibility of achieving GTR. Grade IV tumors have a 0% rate of GTR. Nevertheless, surgical resection with the routine use of intraoperative neuromonitoring remains the first line of treatment. GTR should be sought, if reasonable. Second-line treatment strategies, including radiation and chemotherapy, have demonstrated efficacy as adjuvant therapies to varying degrees. Radiation at an optimal dose of at least 45 Gy has been shown to improve survival. However, the benefit is primarily for those with lower-grade tumors. Differing and novel chemotherapeutic agents have shown promise, but due to the limited number of patients with these tumors and virtual inability to perform prospective studies, it is difficult to delineate if one agent is superior to another. Several human proteins and genes have been implicated in the development of spinal cord astrocytomas and further investigation into the exact biochemical structure and nature of spinal cord astrocytomas would be beneficial in the advancement of treatment options. Due to the significant morbidity associated with attempted or successful resection of astrocytomas, further research on second-line treatment modalities must be pursued to aid in the prevention of disease progression.
